# Assessing the efficacy of a modified crushing technique for the management of concha bullosa: a cone beam computer tomography study

**DOI:** 10.1016/j.bjorl.2020.12.012

**Published:** 2021-01-20

**Authors:** Alireza Mesbahi, Najmeh Movahhedian, Fatemeh Akbarizadeh, Amir A. Hakimi, Leila Khojastepour

**Affiliations:** a Facial Plastic Surgery Clinic, Fars Province, Shiraz, Iran; b Shiraz University of Medical Sciences, Department of Oral and Maxillofacial Radiology, Shiraz, Iran; c Beckman Laser Institute and Medical Clinic, Irvine, United States

**Keywords:** Concha bullosa, Crushing, Technique, Cone beam computed tomography

## Abstract

**Introduction:**

Although many surgical techniques exist to manage obstructive concha bullosa, there continues to be a drive to find the least invasive technique with the fewest complications and best results.

**Objectives:**

The purpose of this study is to describe and assess the short- and long-term efficacy of a modified crushing technique for concha bullosa management.

**Methods:**

Patients who met inclusion criteria underwent a detailed nasal examination and cone beam computed tomography imaging prior to and after septoplasty with crushing surgery for obstructive concha bullosa. Patients were divided into short- and long-term groups based on their followup period such that the short-term group had a mean followup of 15.14 months (range 6–22 months) and the long-term group had a mean followup of 56.66 (range 29–80) months.

**Results:**

Twenty-four cases of obstructive concha bullosa were included in this study with 13 short-term and 11 long-term follow-ups. All patients showed a significantly decreased postoperative CB size (*p* < 0.001). There was no correlation between age and postoperative CB change in area (*p* = 0.39) and no significant difference in the amount of postoperative CB area reduction between the short-term and long-term groups (*p* = 0.35). No patients experienced bleeding, synechia, conchal destruction, or olfactory dysfunction on followup evaluations.

**Conclusions:**

Our modified crushing technique is a simple, effective, and lasting treatment option for concha bullosa. From our experience, there have been no complications and no instances of concha bullosa reformation during the follow-up period.

## Introduction

Middle turbinate pneumatization, termed concha bullosa (CB), is one of the most common sinonasal anatomical variations, reportedly affecting between 14%–53.6% of the population.^1^ Although generally an incidental finding, CB can lead to changes in mucociliary activity and osteomeatal narrowing responsible for maxillary sinus disease, nasal septal deviation, and rhinogenic headache.^2^, ^3^, ^4^, ^5^, ^6^ Consequently, otolaryngologists have long searched for an effective CB treatment.

There is currently no single “gold standard” treatment for CB. Rather, several operative techniques have been previously described including total resection, lateral or medial partial resection, turbinoplasty, and crushing.^6^ Although aggressive resectional techniques improve visualization of the paranasal sinuses, they may result in loss of surgical landmarks, synechia formation, and destabilization of the middle turbinate secondary to extreme surgical manipulation of bone and mucosa.^7^, ^8^, ^9^ Turbinoplasty effectively treats CB and decreases the risk for synechia formation, but it is a more technically challenging procedure that may result in mucosal injury. As such, crushing has become an increasingly popular treatment option.

Crushing is a more conservative and simple treatment for CB that preserves the mucosa. Despite previous reports demonstrating the benefits of crushing technique to treat CB,^3^, ^5^, ^10^ its efficacy and the potential for CB reformation following crushing surgery has been called into question.^8^, ^11^ We modified the crushing technique in order to minimize complications and improve outcomes through a technically straightforward procedure. Herein, we aim to describe this technique and evaluate both its short- and long-term efficacy in the management of CB.

## Methods

The protocol of this study was approved by the Ethical Committee of Shiraz University of Medicine Sciences (protocol number IR.SUMS.REC.1398.498).

### Study design

Patients scheduled for septoplasty with concurrent CB surgery between June 2012 and December 2018 were included in this cross-sectional study. Indications for CB surgery included chronic rhinosinusitis unresponsive to medical treatment. Those who had undergone prior sinonasal surgery were excluded from analysis. All patients underwent a detailed nasal examination and cone beam computed tomography (CBCT) imaging prior to surgery. A second CBCT examination and clinical reassessment were performed as part of patients’ postoperative follow-up.

The mean followup period for patients included in the study was 34.88 months (range 8–80 months). Patients were also divided into short- and long-term groups based on their followup period such that the short-term group had a mean followup of 15.14 months (range 6–22 months) and the long-term group had a mean followup of 56.66 (range 29–80) months.

### CBCT imaging

Both pre- and postoperative CBCT examinations were performed at the same radiology center using the VGI evo NewTom ENT imaging system (QR S.R.L Company, Verona, Italy) at 0.3 mm voxel size, 110 kVp, 7.56 mAS, and a standard field of view.

**Figure 1:**
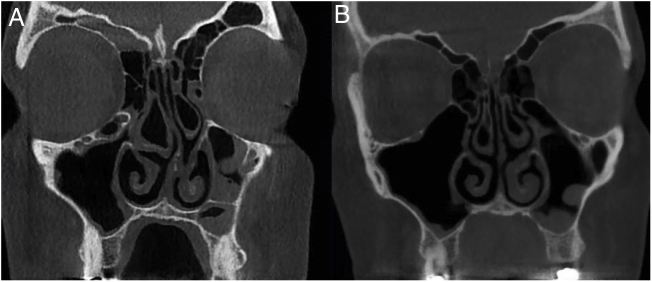
Mean percent reduction of concha bullosa after modified crushing technique among various dimensional parameters.

All radiographs were evaluated concurrently by two oral and maxillofacial radiologists in coronal and axial planes with slice thickness and slice intervals of 1 mm using NNT Viewer software (NNT 9.21, Image Works, Verona, Italy) (Fig. 1). Measurements in the axial plane included: the most anterior-posterior CB dimension, most medio-lateral CB dimension, and maximum CB area. Measurements in the coronal plane included: maximum CB height, most medio-lateral CB dimension, and maximum CB area.

### Surgical technique

Under general anesthesia, the nose was prepared with cottonoid pledgets soaked in oxymetazoline solution. No other injections were required at the CB site. Under endoscopic view, a horizontal incision was made with a number 12 blade along the inferior aspect of the CB from a posterior to anterior direction. A vertical incision was then made with a number 12 blade along the anterior aspect of the CB, meeting the horizontal incision to form an “L”. Both incisions were infiltrated through mucosa and underlying bone. The medial wall of the CB was then lateralized using a blunt instrument, such as a blunt perichondrial elevator. No dressings were required upon completion.

### Statistical analysis

Data analysis was performed using the Statistical Package for the Social Sciences (SPSS) version 17.0 (SPSS Inc., Chicago, IL, USA). A Pearson correlation test was used to assess correlation between CB dimensional changes with age and follow-up duration. Student’s *t*-test was used to compare dimensional parameters before and after the operation and for comparison between the short-term and long-term groups; p-values less than 0.05 were considered to be statistically significant.

## Results

Thirteen patients (eleven with bilateral CB, two with unilateral CB, totaling 24 CB cases) were included in this study. The patients’ average age was 27.5 ± 4.91 years. The short-term followup group included 13 cases of CB and the long-term group included 11 cases. No patients in either group presented with bleeding, synechia, or conchal destruction, during follow-up evaluations.

**Figure 2:**
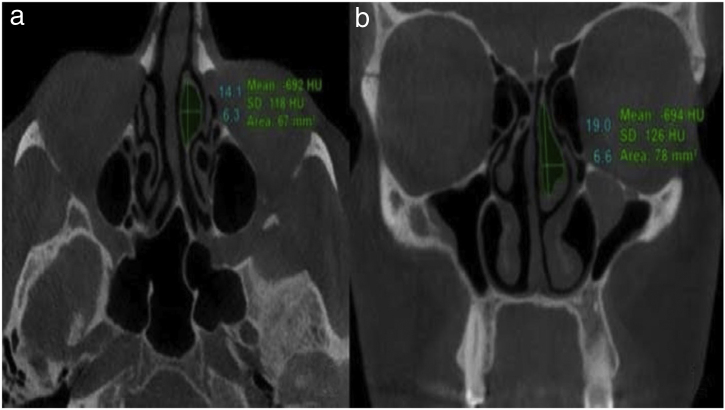
Cone-beam computed tomography images demonstrating (a) anterior-posterior and medio-lateral dimensions as well as overall area in axial plane and (b) height and medio-lateral dimensions in coronal plane.

The average axial and coronal dimension measurements before and after surgery are illustrated in Table 1. All patients showed a significantly decreased post-operative CB size (*p* < 0.001). There was no correlation between age and post-operative CB change in area (*p* = 0.39) (Table 2). There was no significant difference in the amount of CB area change postoperatively between the short- and long-term groups (*p* = 0.35) (Table 3). The greatest amount of mean dimensional reduction seen postoperatively occurred in either the axial or coronal planes followed by the mediolateral plane (Fig. 2).

**Table 1:** Comparison of concha bullosa dimensions before and after modified crushing surgery.

		Pre-operative (Mean ± SD)	Post-operative (Mean ± SD)	*p*-value^a^
Axial	Anterior-posterior dimension (mm)	14.40 ± 6.15	12.83 ± 6.22	< 0.001
	Medio-lateral dimension (mm)	7.01 ± 2.92	4.50 ± 1.80	< 0.001
	Maximum area (mm^2^)	76.48 ± 54.51	45.29 ± 27.91	< 0.001
Coronal	Maximum height (mm)	14.76 ± 7.22	12.39 ± 6.97	< 0.001
	Medio-lateral dimension (mm)	7.17 ± 3.45	4.27 ± 2.30	< 0.001
	Maximum area (mm^2^)	72.27 ± 53.71	40.26 ± 34.04	< 0.001

a Student’s *t*-test.

**Table 2:** Correlation between the concha bullosa dimensional changes and patient's age at the time of operation and at follow-up.

		*p-*value^a^	
		Age at operation	Follow-up
Axial	Anterior-posterior dimension (mm)	0.957	0.857
	Medio-lateral dimension (mm)	0.765	0.243
	Maximum area (mm^2^)	0.917	0.319
Coronal	Maximum height (mm)	0.691	0.963
	Medio-lateral dimension (mm)	0.105	0.386
	Maximum area (mm^2^)	0.308	0.393

a Pearson correlation.

**Table 3:** Comparison of concha bullosa dimensional changes between short-term and long-term follow-up periods.

		Short-term	Long-term	*p*-value^a^
Axial	Anterior-posterior dimension (mm)	15.53 ± 8.93	12.07 ± 10.79	0.400
	Medio-lateral dimension (mm)	31.14 ± 16.39	38.66 ± 12.86	0.231
	Maximum area (mm^2^)	36.45 ± 15.65	40.09 ± 12.86	0.545
Coronal	Maximum height (mm)	18.46 ± 19.32	17.64 ± 12.94	0.906
	Medio-lateral dimension (mm)	3.60 ± 2.14	4.25 ± 1.76	0.433
	Maximum area (mm^2^)	42.39 ± 20.56	50.35 ± 18.83	0.351

a Student’s *t*-test.

## Discussion

In this study, we introduced a novel modification of the crushing technique and provided radiologic evidence using CBCT that confirms CB size significantly decreases after surgery over both the short- and long-term periods. Importantly, none of the patients included in our study experienced postoperative complications like synechia formation, conchal destruction, or CB reformation. As such, our data supports that this technique is an effective long-term treatment for CB.

Our modified crushing technique provides several advantages over resection techniques to diminish both middle and superior bullous turbinates’. It allows the turbinate size to be more carefully manipulated such that the middle meatus can be opened to the appropriate degree. Its conservative nature circumvents excess removal of the normal bony walls and ciliated mucosa of the middle meatus commonly associated with more aggressive resection techniques. Finally, it greatly lessens the risk for cerebrospinal fluid leakage that has been reported with CB resection techniques.^12^

We also demonstrated both the short- and long-term efficacy of this technique. Our short-term data (mean follow-up 15.14 months) is consistent with previous works on crushing technique, demonstrating the significant regression of CB size devoid of CB reoccurrence within a 2 year period.^3^, ^5^, ^10^ Our long-term data (mean followup 56.66 months) provides a longer followup timeframe than any prior studies following the crushing technique, demonstrating that the effects of this technique are long-lasting and devoid of repneumatization. This is an improvement over the crushing technique which, although generally effective, has been reported to result in CB reformation over time.^8^, ^11^ Nevertheless, our results should be received with caution as our sample size was limited. Accordingly, we encourage continued postoperative monitoring of CB patients to ensure long-term symptom relief.

Although computed tomography (CT) is considered the “gold standard” imaging modality for paranasal sinuses, we used CBCT to measure pre- and postoperative CB size. CBCT has several advantages over traditional CT imaging including lower radiation dose, higher resolution, and lower scanning time.^13^, ^14^, ^15^ Additionally, it provides three-dimensional cross-sectional images which offer objective non-distorted visualization of the nasal cavity, unlike two-dimensional imaging platforms which rely upon an operator to estimate geometry based upon a two-dimensional projection of a true three-dimensional surface. CBCT has been previously demonstrated to be highly accurate and reproducible in maxillofacial imaging, using linear measurements of axial and coronal planes.^16^ To further confirm objective measurements of each area, all radiographs in our study were analyzed by two oral and maxillofacial radiologists who provided measurements based on consensus.

We also investigated the impact of age on our modified crushing surgery outcomes. Busaba previously reported that age may impact symptomatic improvement in patients following paranasal sinus surgery.^17^ Specifically, it was shown that younger patients reported olfaction improvement whereas older patients had reported improved rhinorrhea. Age did not significantly impact objective changes in CB area over time following the modified crushing technique among our study sample, suggesting that this technique may be effective regardless of age. However, we did not explore subjective parameters in patient symptoms and our patient population had a more limited age range (18–39 years of age) compared to Basuba’s study population. Future prospective studies are currently underway to correlate objective changes in CB size following the modified crushing technique with subjective changes in patient- reported outcome measures.

## Conclusion

Our modified crushing technique is a safe and conservative treatment option for obstructive CB. Through the use of CBCT radiographs, we were able to demonstrate a long-term significant decrease in CB size following surgery without reformation. Future studies are required to correlate patient symptom improvement with the decreased CB size. This procedure should be considered in lieu of more aggressive CB management practices when applicable.

## Funding

The authors would like to thank the Vice Chancellor for Research, Shiraz University of Medical Science for supporting this investigation (grant nº 18136).

## Ethics committee approval

The protocol of this study was approved by the Ethical Committee of Shiraz University of Medicine Sciences (protocol nº IR.SUMS.REC.1398.498). Informed consent was obtained from all individual participants included in the study. Patients signed informed consent regarding publishing their data and radiographs.

## Conflicts of interest

The authors declare no conflicts of interest.
